# Long non-coding RNA Loc554202 induces apoptosis in colorectal cancer cells via the caspase cleavage cascades

**DOI:** 10.1186/s13046-015-0217-7

**Published:** 2015-09-11

**Authors:** Jie Ding, Binbin Lu, Jianping Wang, Juan Wang, Yongguo Shi, Yifan Lian, Ya Zhu, Jirong Wang, Yingrui Fan, Zhaoxia Wang, Wei De, Keming Wang

**Affiliations:** Department of Oncology, Second Affiliated Hospital, Nanjing Medical University, Nanjing, Jiangsu PR China; Department of clinical laboratory, Second Affiliated Hospital, Nanjing Medical University, Nanjing, Jiangsu PR China; Department of Biochemistry and Molecular Biology, Nanjing Medical University, Nanjing, Jiangsu 210029 PR China

**Keywords:** Loc554202, Apoptosis, Colorectal cancer

## Abstract

**Background:**

Aberrant expression of long noncoding RNAs (lncRNAs) has frequently been reported in cancer studies, including those of colorectal cancer (CRC). Increasing evidence suggests that lncRNAs are significantly correlated with the pathogenesis, development and metastasis of cancer. Loc554202 is a 2166-bp transcript on human chromosome 9p21.3, the expression of which is dysregulated in breast and lung cancer cells. However, its role in CRC remains under investigation.

**Methods:**

Quantitative real-time polymerase chain reaction (qRT-PCR) was carried out to assess the relative expression of Loc554202 in CRC cell lines and tissues. Gain and/or loss of function approaches were used to investigate the potential functional roles in cell proliferation and apoptosis *in vitro* and *in vivo*. qRT-PCR, western-blotting and immunohistochemistry were used to evaluate the mRNA and protein expression of apoptosis-related factors.

**Results:**

Loc554202 was significantly downregulated in cancerous tissues and CRC cell lines compared with adjacent normal tissue and a normal human intestinal epithelial cell line. Low Loc554202 expression was closely associated with advanced pathologic stage and a larger tumor size. The overexpression of Loc554202 decreased the cell proliferation and induced apoptosis *in vitro* and hindered tumorigenesis *in vivo*. Loc554202 regulated cell apoptosis partly through the activation of specific caspase cleavage cascades.

**Conclusion:**

Our results suggest that Loc554202 may play an important role in the progression of CRC and could be a candidate prognostic biomarker or a target for new cancer therapies.

**Electronic supplementary material:**

The online version of this article (doi:10.1186/s13046-015-0217-7) contains supplementary material, which is available to authorized users.

## Introduction

Colorectal cancer (CRC), as the third most common cancer affecting the gastrointestinal tract, is a common type of cancer worldwide, and is associated with a high mortality rate due to its rapid progression and advanced tumor presentation at the time of diagnosis [1–3]. CRC is becoming more prevalent in China [4]. The vast majority of CRC patients have reached an advanced pathological stage before symptoms appear; therefore, that the survival rate of those diagnosed with CRC remains poor. The early detection of CRC is significantly beneficial to improve the probability of survival. Therefore, a better understanding of the mechanisms that result in CRC and the identification of a new molecular marker or factor that can be used to create novel diagnostic and therapeutic strategies is urgently needed for patients with CRC.

Long non-coding RNAs (lncRNAs, > 200 nucleotides in length) are a class of newly discovered non-coding RNA molecules with limited or no protein-coding capacity [5, 6]. Recently, increasing evidence has shown that lncRNAs have important biological functions are closely related to human diseases, especially cancer [7–10]. They may function as oncogenes [11, 12] or anti-oncogenes [13, 14] similar to protein-coding genes, and their dysregulated expression is significantly correlated with carcinogenesis [15]; therefore, they may be considered to be promising candidate biomarkers for diagnosing cancer and may also represent therapeutic target in the future.

The underlying molecular mechanisms by which lncRNAs exert their functions are complex, diverse and exist at various levels during the development of cancer, including the epigenetic, transcriptional, post-transcriptional and translational levels [16–18]. For instance, lncRNA HOTAIR is upregulated in CRC and may be a critical element in metastatic progression as a result of its interaction with PRC2 (Polycomb Repressive Complex 2) [19]. Our previous studies also showed that HOTAIR could function as a competing endogenous RNA to regulate HER2 expression by sponging miR-331-3p in gastric cancer [20]; lncRNA SPRY4-IT1 could be a growth regulator, and promoted the proliferation of ER (−) human breast cancer cells by upregulating the expression of ZNF703 [21].

Loc554202, is a 2166-bp transcript on human chromosome 9p21.3, which is the host gene of miR-31 and dysregulated in breast [22, 23] and lung [24] cancer cells, although the importance of its function has not yet been established. Little is known concerning the potential role of Loc554202 in the development and progression of CRC. To detect the functions and molecular mechanisms of Loc554202 in CRC, we conducted a qRT-PCR analysis of Loc554202 expression in human CRC tissues and cell lines, and showed that the expression of Loc554202 was significantly downregulated in cancerous tissue samples and CRC cell lines compared with matched normal samples. Its downregulation was closely associated with an advanced pathological stage and a large tumor size. Further functional studies of Loc554202 indicated that enhanced expression of Loc554202 could decrease cell proliferation and induce apoptosis *in vitro* and *in vivo*.

## Materials and methods

### Tissue collection

Colorectal cancer tissues and normal tissues were obtained from 48 patients who had undergone surgical resection of colorectal cancer between 2010 and 2012 at Second Affiliated Hospital of Nanjing Medical, China. No local or systemic treatment had been administered to these patients prior to the operation. All tissue samples were washed with sterile phosphate-buffered saline before being snap frozen in liquid nitrogen and then stored at −80 °C until required for the analyses. The pathological stage, grade and nodal status were appraised by an experienced pathologist. The clinicopathological characteristics, including the tumor, node metastasis (TNM) staging are summarized in Table 1. The non-tumorous tissues were 5 cm from the edge of the tumor, and there were no obvious tumor cells present in these regions as determined by the pathologist. All of the experiments were approved by the Research Ethics Committee of Nanjing Medical University, China. Written informed consent was obtained from all patients.

**Table 1:** Correlation between Loc554202 expression and clinicopathological characteristics of CRC patients

Characteristics	Number	Percent	Loc554202		*p*
	(*n* = 48)		High	Low	Chi-squared test *P*-value
Age (years)					0.560
<60	21	43.8 %	11	10	
≥60	27	56.2	13	14	
Gende					0.430
Male	29	60.4 %	15	14	
Female	19	39.6 %	9	10	
Maximum tumor size					0.007*
<5 cm	28	58.3 %	19	9	
≥5 cm	20	41.7 %	5	15	
Location					0.440
Colon	19	39.6 %	10	9	
Rectum	29	60.4 %	14	15	
Depth of tumor					0.108
T1 and T2	18	37.5 %	11	7	
T3 and T4	30	62.5 %	13	17	
Tumor stage					0.028*
I and II	26	54.2 %	17	9	
III and IV	22	45.8 %	7	15	
Lymph node metastasis					0.698
Negative	25	52.1 %	12	13	
Positive	23	47.9 %	12	11	

* *P*<0.05 was considered significant (Chi-square test between two groups)

### Ethics statement

The study was approved by the Ethics Committee of Nanjing Medical University and was performed in compliance with the Declaration of Helsinki. Written informed consent was obtained for the use of all patient samples. All experimental animals were housed under specific pathogen-free conditions. All experimental procedures were approved by the Institutional Review Board of Nanjing Medical University. All procedures were performed in accordance with the Nanjing Medical University Guide for the Care and Use of Laboratory Animals which was formulated by the National Society for Medical Research.

### Cell lines and culture conditions

The human colorectal cancer cell lines (HCT116, DLD1, SW480, RKO, HT-29) were purchased from the Institute of Biochemistry and Cell Biology of the Chinese Academy of Sciences (Shanghai, China). The human colonic epithelial cells HCoEpiC were obtained from American Type Culture Collection (Manassas, VA). They were cultured in Dulbecco’s modified Eagle’s medium (DMEM; Invitrogen) in humidified air at 37 °C with 5 % CO2. All media were supplemented with 10 % fetal bovine serum (10 % FBS), 100 U/ml penicillin, and 100 mg/ml streptomycin (Invitrogen, Shanghai, China).

### RNA extraction and qRT-PCR analyses

Total RNA was extracted from tissues or cultured cells with the TRIZOL reagent (Invitrogen Life Technologies) according to the manufacturer’s protocols. For qRT-PCR, RNA reverse transcribed to cDNA from 1 μg of total RNA was reverse transcribed in a final volume of 20 μl using random primers and a Reverse Transcription Kit (Takara, Dalian, China). According to the manufacturer’s instructions, the reverse transcription was performed at 37 °C for 15 min, then at 85 °C for 5 s. qRT-PCR analyses were performed using a standard protocol from Power SYBR Green (Takara, Dalian, China). All protocols were performed according to the manufacturer’s instructions. The Δct values were normalized to those of glyceraldehyde-3-phosphate dehydrogenase (GAPDH). The primer sequences used for the studies are shown in Additional file 1: Table S1. The qRT-PCR assays and data collection were performed using an ABI 7500 instrument. Each sample was analyzed in triplicate.

### Treatment of HCT116 and DLD1 cells with 5-aza-2-deoxy-cytidine (5-aza-CdR)

CRC cells (2.5 × 105) were seeded into six-well cultureplates and exposed to 0, 5 or 10 μM 5-aza-CdR (Sigma-Aldrich, USA). Cells were harvested after 72 h for qRT-PCR to detect the expression level of Loc554202.

### Transfection of colorectal cancer cells

Small interfering RNA (siRNA) and nonspecific control siRNA were synthesized (Carlsbad, California, USA) and transfected into cells using Lipofectamine 2000 (Invitrogen, USA). To overexpress Loc554202, the full length coding sequence for Loc554202 was amplified and subcloned into the pcDNA 3.1(+) vector (Invitrogen) according to the manufacturer’s instructions. HCT116 and DLD1 cells were transfected with a negative control vector or the Loc554202-expressing plasmid according to the manufacturer’s protocol. Cells were harvested after 48 h for qRT-PCR and western blot analyses. The sequences of the siRNAs are described in Additional file 2: Table S2.

### Cell proliferation assays

Forty-eight hours after pCDNA-Loc554202 transfection, 3,000 cells per well were allowed to grow in 96-well plates with five replicate wells. After 6 h of culture, as well as at 24, 48, 72 and 96 h after atarting the culture, the cells were treated with 100 μg 3-(4,5-dimethylthiazol-2-yl)-2,5-diphenyltetrazolium bromide (MTT) by adding it to the medium. The cells were incubated at 37 °C for another 4 h, then the medium was removed, and dimethylsulfoxide (DMSO) was added for 10 min to lyse the cells. Finally, the absorbance was measured at 490 nm. All experiments were performed in triplicate.

### Colony formation and clonogenic assays

Cells were trypsinized into single-cell suspensions 48 h after transfection. For the colony formation assay, 1,000 cells were plated into each well of a six-well plate and were maintained in media containing 10 % FBS to allow colony formation, with the medium being repalaced every four days. After two weeks, colonies were fixed with methanol and stained with 0.1 % crystal violet (Sigma) in PBS for 15 min. The visible colonies were manually counted. Triplicate wells were measured for each treatment group.

### Flow cytometry

Cells transiently transfected with pCDNA-Loc554202 were harvested 48 h after transfection by trypsinization, washed with ice-cold phosphate-buffered saline, and fixed with 75 % ethanol overnight. The cells used for the cell-cycle analysis were stained with propidium oxide (100 μg/mL) using the Cycle Test Plus DNA Reagent Kit (BD Biosciences) and were analyzed by flow cytometry (FACScan;BD Biosciences) using an instrument equipped with the CellQuest software program (BD Biosciences). The percentages of cells in the G0–G1, S, and G2–M phases were counted and compared. The cells used for the apoptosis analysis were harvested 48 h after transfection, and were stained for 15 min with fluorescein isothio-cyanate (FITC)-Annexin V and propidium iodide (PI) in the dark at room temperature, according to the manufacturer’s recommendations. The cells were then examined by flow cytometry (FACScan; BD Biosciences) and the CellQuest software program (BD Biosciences) and were discriminated into viable cells, dead cells, early apoptotic cells, and apoptotic cells. The percentage of early apoptotic cells was compared with the control groups from each experiment.

All of the samples were assayed in triplicate.

### TUNEL

Terminal deoxynucleotidyl transferase-mediated dUTP nick end labeling (TUNEL) was performed with an apoptosis detection kit (KeyGEN BioTECH, China) according to the manufacturer’s instructions. Randomly selected fields without significant necrosis in 10 high-power fields (6400) were assessed for TUNEL-positive cells. The TUNEL index was calculated based on the number of total nuclei and the number of cells with green nuclei.

### Western blot analysis and antibodies

Cells were lysed using the mammalian protein extraction reagent, RIPA (Beyotime), supplemented with a protease inhibitor cocktail (Roche) and PMSF (Roche). Protein were separated by 10 % sodium dodecyl sulfate–polyacrylamide gel electrophoresis (SDS–PAGE) and transferred to 0.22 mm nitrocellulose (NC) or polyvinylidene difluoride membranes (Sigma). The membranes were washed, blocked, and incubated with specific primary antihuman antibodies. The secondary antibody was horseradish peroxidase-conjugated goat anti-rabbit IgG. An ECL chromogenic substrate was used to visualize the bands and the intensity of the bands was quantified by densitometry (Quantity One software; Bio-Rad). The mean ± SD values were calculated from three individual experiments. A GAPDH antibody was used as a control, and the anti-cleaved caspase-3, cleaved caspase-9, Bcl-2 and Bax (all 1:1000) antibodies were purchased from Cell Signaling Technology, Inc. (CST).

### Tumor formation assay in a nude mouse model

Four-week-old male BALB/c nude mice were obtained from the Shanghai Laboratory Animals Center of the Chinese Academy of Sciences (Shanghai, China). The mice were housed under pathogen-free conditions with a 12 h light/dark schedule, were fed an autoclaved diet *ad libitum*, and were injected subcutaneously with 5 × 10^6^ cells to assess the tumor formation. Tumor growth was examined every three days, and tumor volumes were calculated using the following formula: 0.5 × length × width^2^. At two weeks after the cell injection, the mice were killed, and the subcutaneous weight of each tumor was measured and the tumors were used for further analyse. The protocol was approved by the Committee on the Ethics of Animal Experiments of the Nanjing Medical University.

### Immunohistochemical (IHC) analysis

Tumor tissue samples were immunostained for cleaved caspase-3 as described previously. The expression was considered to be positive when 50 % or more cancer cells were stained. The anti-cleaved caspase-3(1:50) antibody was purchased from CST.

### Statistical analysis

All data were expressed as the means ± SD (standard deviation, SD), and were analyzed using Student’s *t* test between to compare two groups of *in vitro* and *in vivo* data using the SPSS 17.0 software program. A value of *P* < 0.05 was considered to be statistically significant.

## Results

### Loc554202 expression is downregulated in human colorectal cancer tissues and colorectal cancer cell lines

**Fig. 1:**
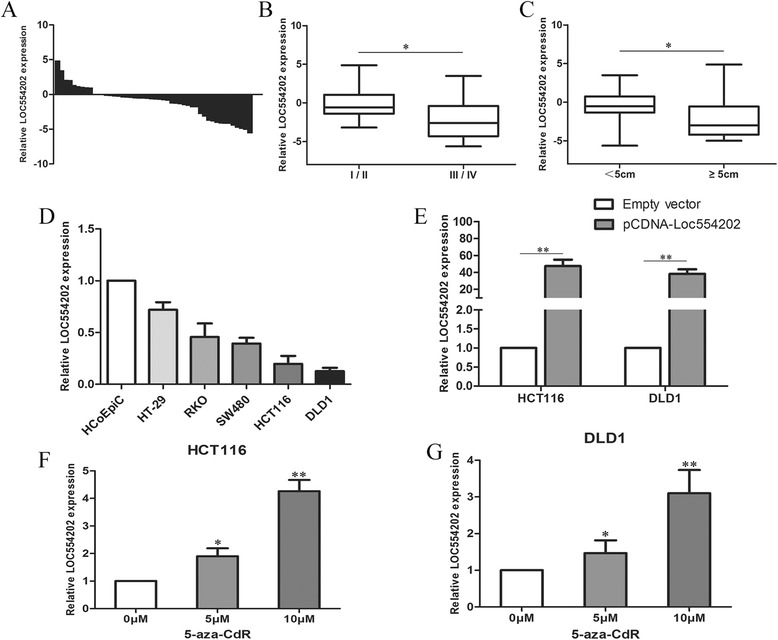
Relative expression of Loc554202 in colorectal cancer tissues and cells compared with adjacent normal tissues and normal colonic epithelial cells. **a** The relative expression of Loc554202 in colorectal cancer tissues (*n* = 48) compared with corresponding non-tumor tissues (*n* = 48). The Loc554202 expression was examined by qRT-PCR and was normalized to the GAPDH expression. The results are presented as the fold-change in tumor tissues relative to normal tissues (shown as -ΔΔCT). **b** and **c**) The data are presented as the relative expression levels in tumor tissues. The Loc554202 expression was significantly lower in patients with a higher pathological stage and larger tumors. **d** The Loc554202 expression was assessed by qRT-PCR in colorectal cancer cell lines (HT-29, RKO, SW480, HCT116, DLD1) and was compared with the normal human colonic epithelial cell line (HCoEpiC). **e** The relative expression levels of Loc554202 following the treatment of HCT116 and DLD1 cells with pCDNA-Loc554202 and empty vector. (**f** and **g**) The level of Loc554202 expression in HCT116 and DLD1 cells following treatment with 5-aza-dC (0, 5, 10 μM).* *P* < 0.05 and ** *P* < 0.01

We first examined the Loc554202 expression levels in 48 colorectal cancer tissue samples and 48 matched normal colorectal tissue samples by performing a qRT-PCR analysis. The Loc554202 expression was downregulated (*P* < 0.05) in the cancerous tissues compared with the corresponding adjacent non-tumorous tissues (Fig. 1a). To further understand the significance of Loc554202 expression in CRC, we then evaluated the correlation of Loc554202 expression with various clinicopathological features (i.e., stage, maximum diameter) (Table 1). As shown in Fig. 1b and c, low Loc554202 expression in CRC was significantly correlated with the tumor size (*p* = 0.003) and advanced TNM stage (*p* = 0.020), which reflect a higher tumor burden. However, Loc554202 expression was not associated with other parameters such as age (*p* = 0.146) or gender (*p* = 0.768) in CRC patients (Table 1). These results imply that Loc554202 may be a potential prognostic biomarker for CRC patients.

We next examined the expression of Loc554202 in five human colorectal cancer cell lines (HT-29, SW480, RKO, HCT116, and DLD1) and a normal colonic epithelial cell (HCoEpiC). Figure 1d shows that the Loc554202 expression levels were indeed lower in all of the CRC cell lines examined compared with the normal intestinal epithelial cells (*p* < 0.01). We chose to use the HCT116 and DLD1 cell lines for further studies because they had the lowest expression levels. Furthermore, to detect the transfection efficiency and eliminate the influence of miR-31 may exist on overexpression of Loc554202, the expression levels of Loc554202 and miR-31 were detected by a qRT-PCR analysis after transfecting these cells with pCDNA-Loc554202. The results found that the Loc554202 expression was increased by 47.5-fold and 38-fold in the HCT116 and DLD1 cells, respectively, compared with the control cells following transfection with pCDNA-Loc554202 (Fig. 1e), and there was no significant change in miR-31 expression (Additional file 3: Figure S1).

### Effects of DNA methylation on the Loc554202 expression in colorectal cancer cells

Although the hypermethylation of Loc554202 has been reported to be involved in its transcriptional inactivation in breast cancer, whether this is also the case in CRC was unknown. To determin the influence of DNA methylation in CRC cells, we treated HCT116 and DLD1 cells with a DNA demethylating agent (5-aza-CdR) for 72 h, and found that the Loc554202 expression was significantly increased by 1.9- or 4.2- fold, or by 1.4- or 3.1- fold in the 5-aza-CdR-treated HCT116 (Fig. 1f) and DLD1 (Fig. 1g) cells, respectively, compared with the control cells.

### Overexpression of LOC554202 inhibits colorectal cancer cell proliferation *in vitro*

**Fig. 2:**
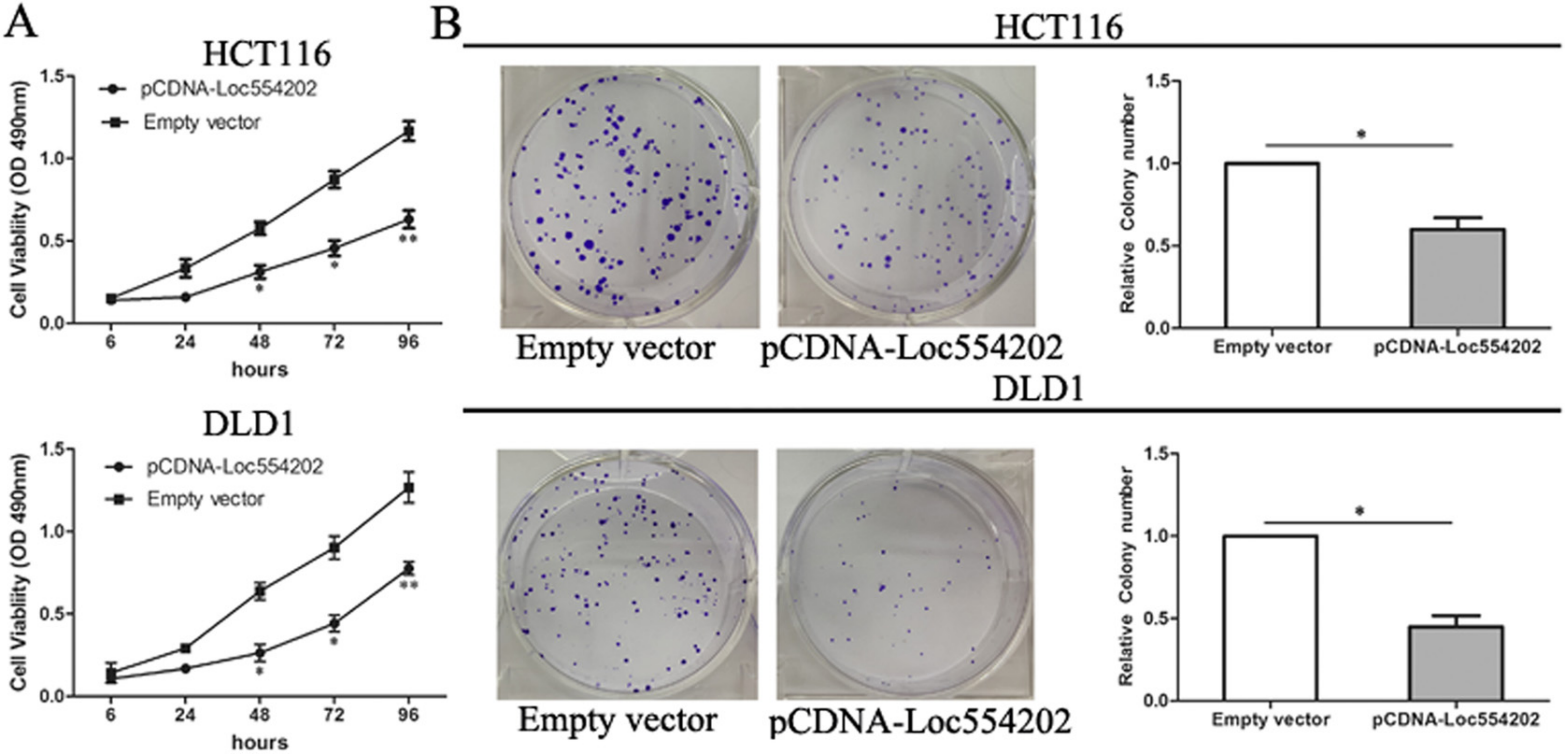
Effects of Loc554202 overexpression on colorectal cancer cell proliferation *in vitro*. **a** A MTT assay was performed to determine the proliferation of HCT116 and DLD1 cells following treatment with pCDNA-Loc554202 or empty vector. The data represent the means ± S.D. from three independent experiments. **b** Colony-forming growth assays were performed to determine the proliferation of HCT116 and DLD1 cells. The colonies were counted and captured. * *P* < 0.05 and ** *P* < 0.01

To investigate the potential biological function of Loc554202 in CRC cells, the MTT assay was performed to detect the impact of Loc554202 overexpression on the proliferation of HCT116 and DLD1 colorectal cancer cells. The results showed that the growth of HCT116 and DLD1 cells transfected with pCDNA-Loc554202 was significantly inhibited compared with that of control cells (*p* < 0.05; Fig. 2a). Similarly, colony formation assays were performed to reveal the long-term anti-proliferative effects of Loc554202 on the growth of HCT116 and DLD1 cells. These data suggest that the colony numbers of colorectal cancer cells transfected with pCDNA-Loc554202 were obviously lower than those of the negative control cells (*p* < 0.05; Fig. 2b). These findings indicate that Loc554202 may act as a tumor suppressor, and may inhibit CRC cell proliferation.

### Overexpression of Loc554202 promotes G1 arrest and causes apoptosis in colorectal cancer cells *in vitro*

**Fig. 3:**
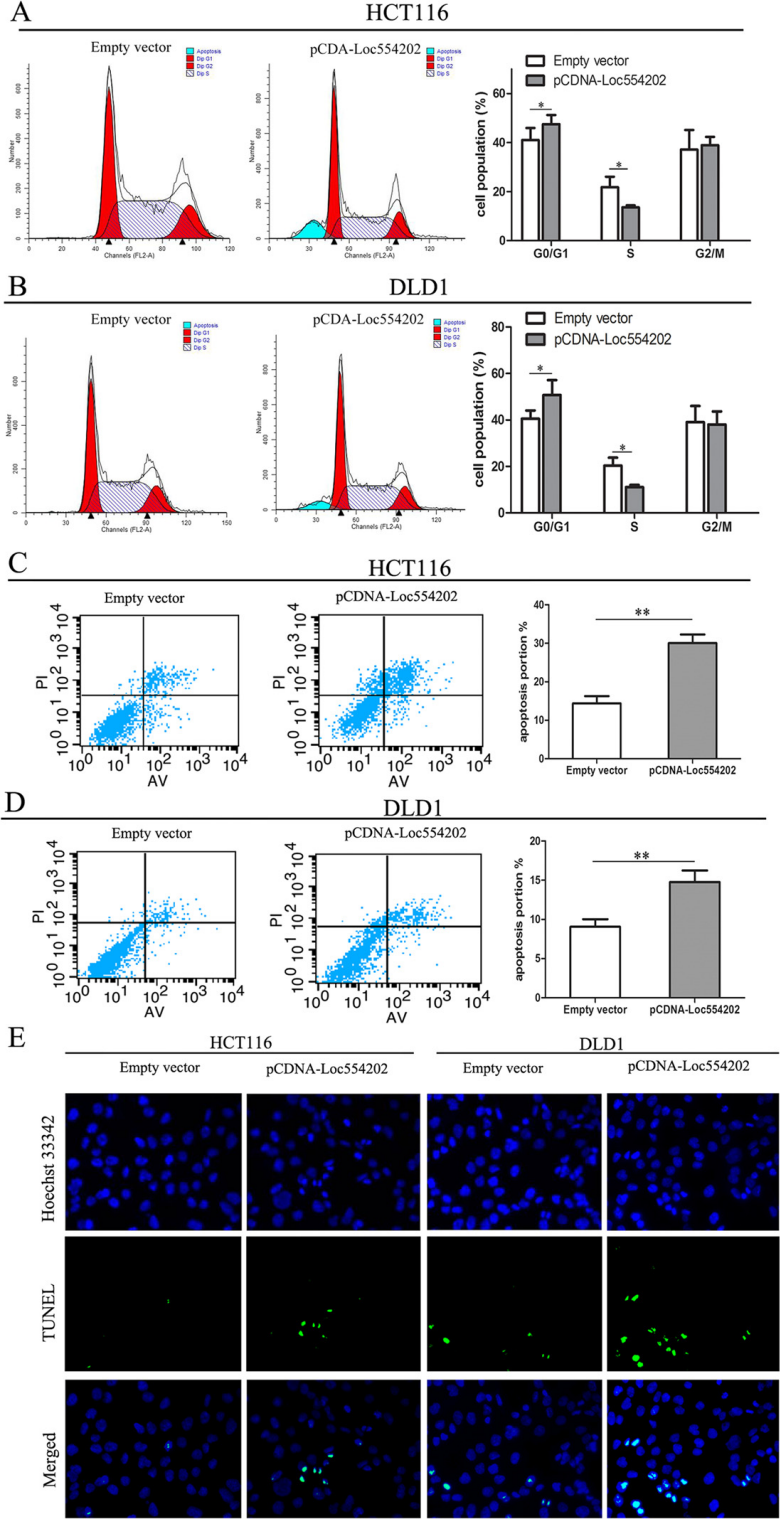
Effects of Loc554202 on the cell cycle and apoptosis of colorectal cancer cells *in vitro.* HCT116 and DLD1 cells were transfected with pCDNA-Loc554202 or empty vector. **a** and **b** The bar chart represents the percentage of cells in the G0/G1, S, or G2/M phases, as indicated. **c** and **d** The percentage of apoptotic cells was determined by a flowcytometric analysis. The data represent the means ± S.D. from three independent experiments. **e** The level of apoptosis in HCT116 and DLD1 cells after they were transfected with pCDNA-Loc554202 or empty vector as determined by TUNEL staining.*P < 0.05

To further assess whether the anti-proliferative effects of Loc554202 on the CRC cells were mediated by inhibiting cell cycle progression, we examined the cell cycling in HCT116 and DLD1 cells by flow cytometry. The results demonstrated that Loc554202 overexpression promoted a significant arrest of both cell lines in the G0/G1-phase (*p* < 0.05), with an obvious reduction in the number of cells in the S-phase (*p* < 0.05; Fig. 3a and b). In addition, to investigate whether apoptosis regulation was potential contributing factor to the cell growth inhibition induced by Loc554202, an apoptosis assay was performed using flow cytometric analysis. As shown in Fig. 3c and d, the proportions of apoptotic cells following pCDNA-Loc554202 treatment were significantly increased compared with those in the control group (*p* < 0.05). Moreover, a microscopic analysis of TUNEL staining in HCT116 and DLD1 cells confirmed these findings (Fig. 3e). Taken together, these data indicate that Loc554202 treatment could arrest cells in the G0/G1-phase of the cell cycle and induce apoptosis in CRC cells. These data suggest that Loc554202 exerts critical effects on CRC cells by affecting both the cell cycle and apoptosis.

### Knockdown of Loc554202 mildly promotes the proliferation of HCT116 and DLD1 cells *in vitro*

**Fig. 4:**
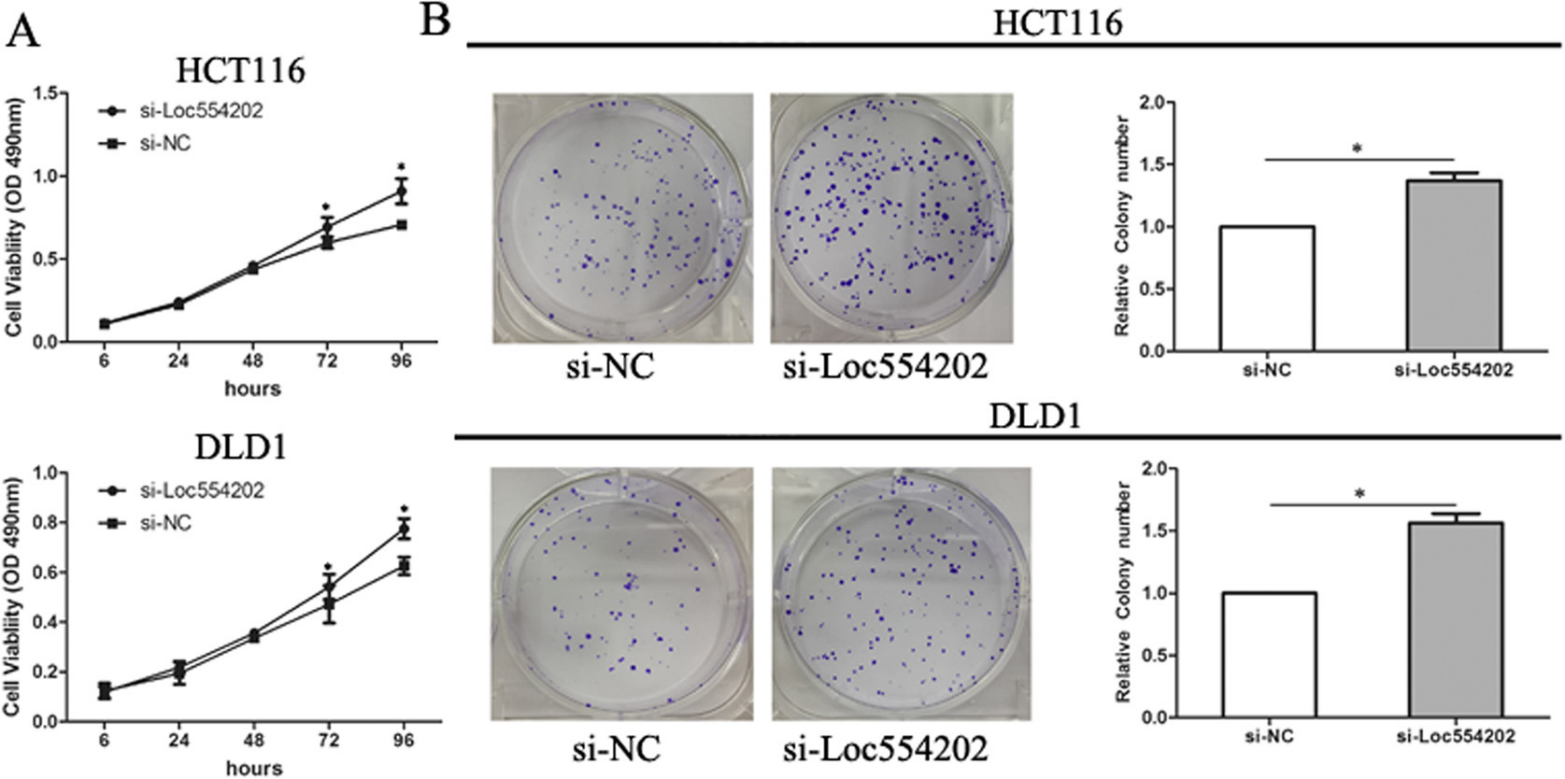
Effects of Loc554202 knockdown on colorectal cancer cell proliferation *in vitro*. **a** A MTT assay was performed to determine the proliferation of HCT116 and DLD1 cells following transfection with si-Loc554202 or si-NC. The data represent the means ± S.D. from three independent experiments. **b** Colony-forming growth assays were performed to determine the proliferation of HCT116 and DLD1 cells. The colonies were counted and captured. * *P* < 0.05 and ** *P* < 0.01

To verify that the Loc554202 expression was inversely related to colorectal cancer progression, we used siRNAs to downregulate the endogenous Loc554202 expression in the HCT116 and DLD1 cell lines. MTT assays and colony formation assays were performed as described previously, and we found that the siRNA transfection-mediated Loc554202 knockdown increased the cell growth compared with negative control cells in both cell lines. The cell viabilities of both cell lines following treatment with the Loc554202 siRNA were moderately higher than that in the control group (*p* < 0.05; Fig. 4a). The results of the colonyformation assay showed that the clonogenic survival was increased when Loc554202 was downregulated in the HCT116 and DLD1 cell lines (*p* < 0.05; Fig. 4b). These observations indicated that low Loc554202 expression may contribute to CRC progression.

### Overexpression of Loc554202 inhibits CRC tumorigenesis *in vivo*

**Fig. 5:**
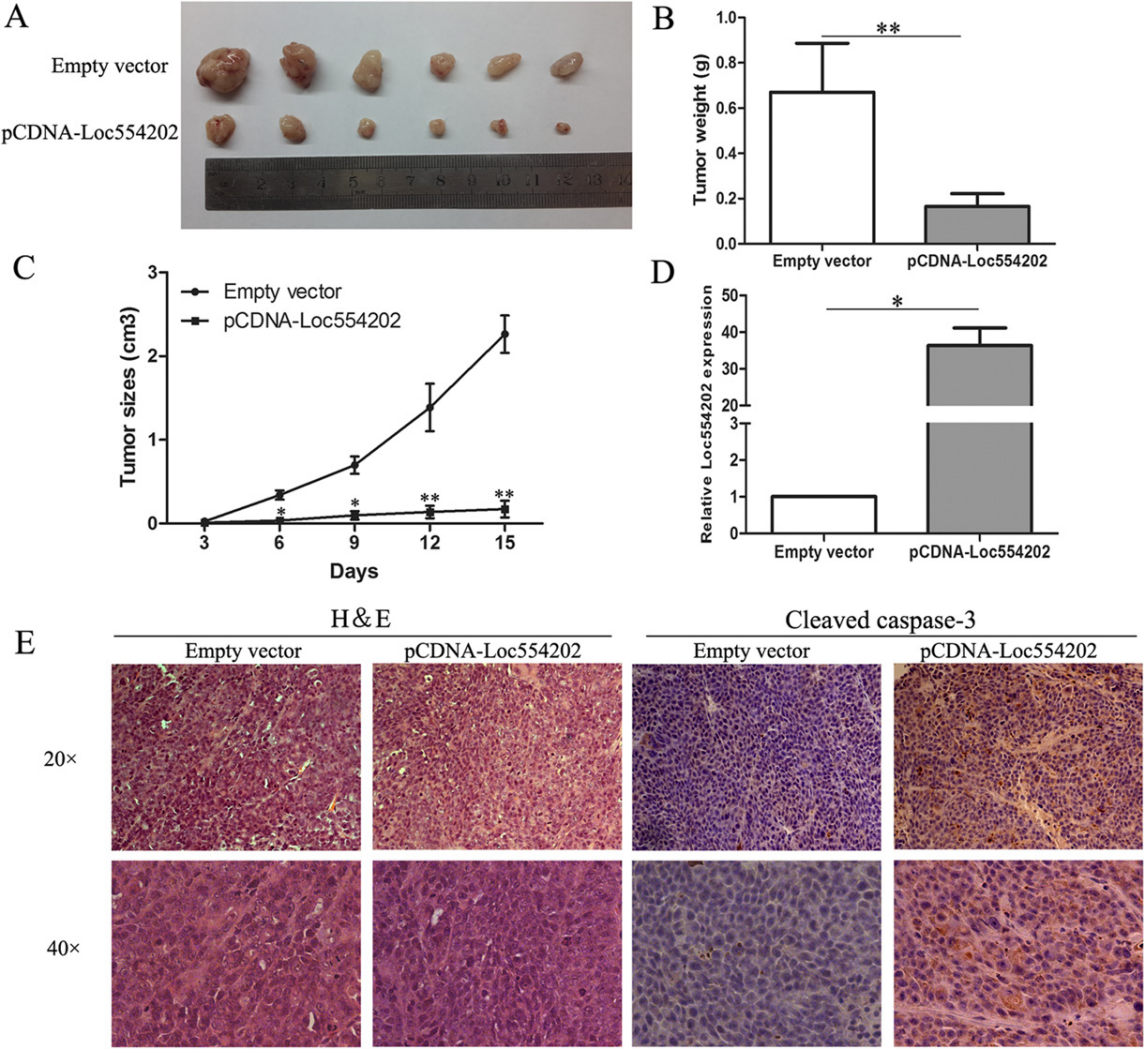
Loc554202 inhibits tumor growth in a xenograft mouse model. **a** The total numbers of tumors after removal from the mice. **b** The tumor weights after the tumors were harvested. The data represent the means ± SD. ** *P* < 0.01. **c** The tumor volumes were calculated every three days after inoculation. **d** qRT-PCR analyses indicated that the Loc554202 expression was significantly increased *in vivo*. **e** Representative images of HE staining and immunohistochemical staining of the tumor. IHC showed an upregulation of the apoptosis index and cleaved caspase-3

To determine whether the overexpression of Loc554202 could affect tumorigenesis, pCDNA-Loc554202 and empty vector transfected HCT116 cells were inoculated into male nude mice. Fifteen days after inoculation, all mice developed xenograft tumors at the injection site. However, the tumors formed in the pCDNA-Loc554202 group were dramatically smaller than those in the empty vector group (Fig. 5a). Remarkably, the average tumor weight was obviously lower in the pCDNA-Loc554202 group compared with the empty vector group (Fig. 5b). Consistently, the tumor growth in the pCDNA-Loc554202 group was significantly slower than that in the control group (Fig. 5c). A qRT-PCR analysis of the Loc554202 expression was then performed using the xenograft tumor tissues. The results showed that the levels of Loc554202 expression in tumor tissues formed from pCDNA-Loc554202 cells were lower than those of the tumors formed in the control group (*p* < 0.05, Fig. 5d). We also examined the HE staining of tumor tissues and found that karyopyknosis and a shape change were present in the tumor samples treated with pCDNA-Loc554202, and these findings were not noted in the negative control samples (Fig. 5e). The tumors developed from pCDNA-Loc554202 cells displayed significantly increased cleaved caspase-3 staining compared with tumors formed from empty vector transfected cells (Fig. 5f). These results indicate that Loc554202 is significantly associated with the proliferation and apoptosis of colorectal cancer cells *in vivo*.

### Loc554202 promotes the apoptosis of colorectal cancer cells via the caspase cleavage cascades

**Fig. 6:**
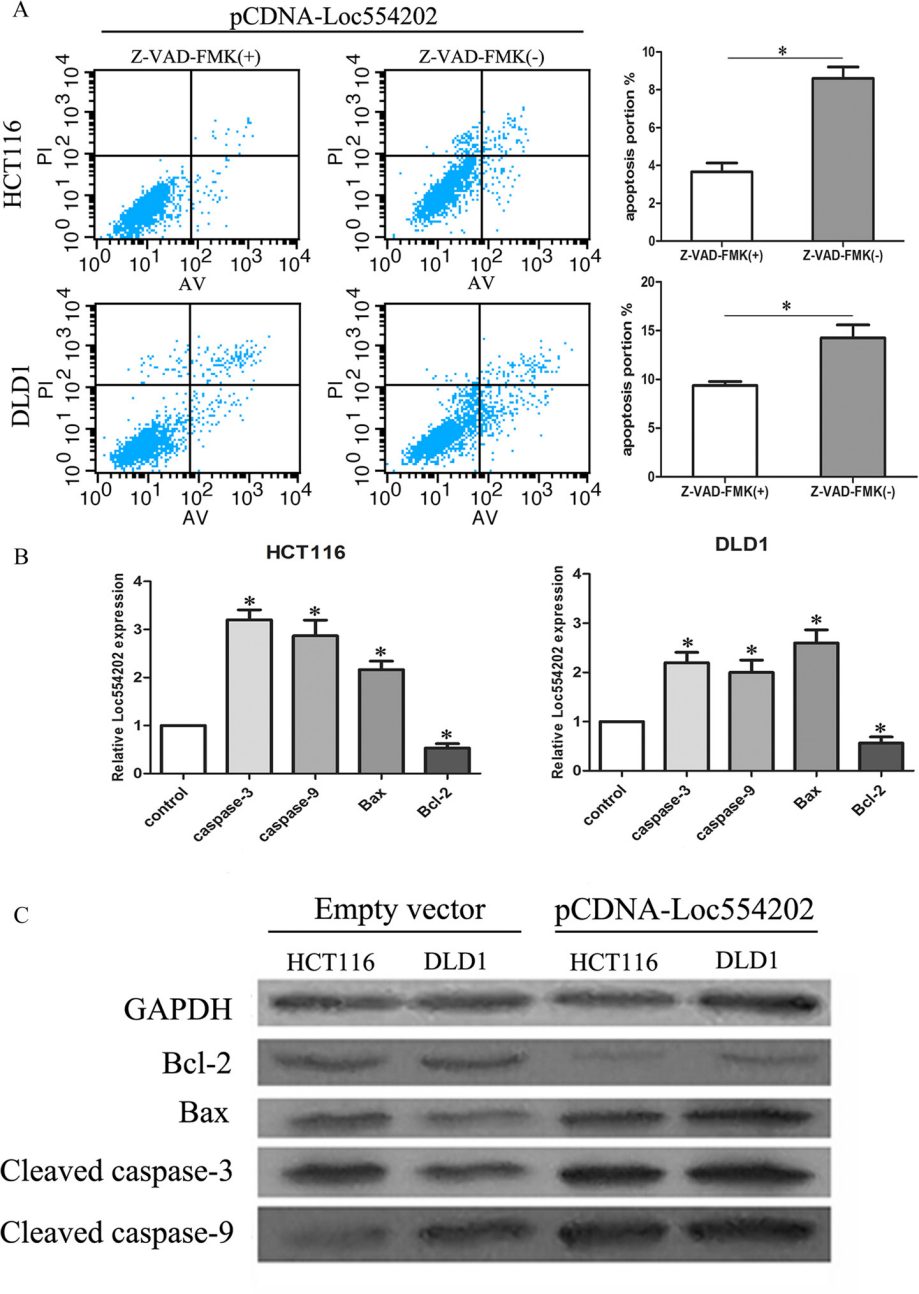
Loc554202 promotes the apoptosis of colorectal cancer cells via the caspase cleavage cascades. **a** HCT116 and DLD1 cells were transfected with pCDNA-Loc554202 in the presence or absence of Z-VAD-FMK (10 mmol/L) for 24 h. The apoptotic status of the cells was determined by a flowcytometric analysis. The data represent the means ± SD. * *P* < 0.05. **b** The results of the qRT-PCR analysis of caspase-3, caspase-9, Bax, and Bcl-2 after the transfection of pCDNA-Loc554202 or empty vector into HCT116 and DLD1 cells. **c** The results of the western blot analysis of the levels of cleaved caspase-3, cleaved caspase-9, Bax and Bcl-2 after the transfection of pCDNA-Loc554202 or empty vector into HCT116 and DLD1 cells. The GAPDH protein was used as an internal control. *P < 0.05

Various mechanisms have been studied to explain lncRNA-mediated cell apoptosis, and the most important pathway identified is the activation of specific caspase cleavage cascades. To confirm the role of caspase activation in Loc554202 induced apoptosis, we treated HCT116 and DLD1 cells with a general caspase inhibitor, Z-VAD-FMK (10 mmol/L). As shown in Fig. 6a, the pretreatment with Z-VAD-FMK decreased the Loc554202 induced apoptosis rate detected by flow cytometry. Consistent with this finding, the results of a qRT-PCR analysis and western blot analysis showed that the mRNA levels of Bax, caspase-3 and caspase-9 and the protein levels of Bax, cleaved caspase-3, cleaved caspase-9 were significantly increased in pCDNA-Loc554202 treated cells, whereas the mRNA and protein levels of Bcl-2 were decreased (Fig. 6b and c). These findings indicate that Loc554202 induces CRC cell apoptosis at least partly through the activation of specific caspase cleavage cascades.

## Discussion

Over the past few decades, accumulating evidence has shown that long non-coding RNAs (lncRNAs) play an important role in the pathogenesis of cancer [6, 25, 26], and can provide a novel platform for the diagnosis and treatment of this disease. With the advances in tiling array and novel sequencing technologies, it has been revealed that only 2 % of the total human genome sequences encode proteins, whereas the remainder can be divided into two groups according length, including short ncRNAs (<200 nt) and long ncRNAs (>200 nt) [27, 28]. The lncRNAs are important new members of the ncRNA family, which are greater than 200 nt in length and can be transcribed by RNA polymerase II (RNA pol II). Recent, evidence has indicated that many lncRNAs can be considered as vital regulators of tumorigenesis [29, 30], as were lncRNA SPRY4-IT1 [21] and HOTAIR [20] in our previous studies. Therefore, the identification of cancer-associated lncRNAs and investigation of their molecular and biological functions are important to provide new insights into the diagnosis and treatment of cancer, including colorectal cancer.

A previous study demonstrated that Loc554202 as the host gene of miR-31 regulates the proliferation and migration of breast cancer cells [22], and CpG island methylation plays an important role in silencing the Loc554202 genes [23]. However, the functions of Loc554202 in CRC were previously unknown. In this study, we provided the first evidence that lncRNA Loc554202 was significantly downregulated in colorectal cancer tissues compared with adjacent normal tissues, and low expression of the lncRNA in CRC patients was associated with an increased tumor size and advanced TNM stage. The expression level of miR-31 was no significant change after transfecting CRC cells with pCDNA-Loc554202, which eliminated the influence of miR-31 may exist on overexpression of Loc554202. Additionally, DNA methylation could restrain the expression of Loc554202. Our subsequent studies showed that overexpression of Loc554202 decreased cell proliferation and caused a dramatic decrease in colony formation in the HCT116 and DLD1 cells. This result was confirmed by knocking down Loc554202 in both CRC cell lines. These findings suggest that Loc554202 might be a novel clinical marker for the prognosis of CRC and might represent a target for therapy.

To further clarify the functions of Loc554202 in CRC, such as whether its expression influenced cell cycle progression and apoptosis, we next used a flow cytometry assay to detect the cell cycle progression and apoptosis in HCT116 and DLD1cells after treatment with pCDNA-Loc554202 or an empty vector. The results demonstrated that Loc554202 overexpression promoted significant arrest in the G0/G1-phase and an obvious increase in apoptosis. These observations were verified by means of TUNEL staining, immunohistochemical (IHC) assays and in a mouse xenograft model. We also found that silencing Loc554202 expression mildly inhibited apoptosis in HCT116 and DLD1 cells *in vitro.* These results revealed that the anti-proliferative effects of Loc554202 in the CRC cells were mediated by its inhibition of cell cycle progression and induction of apoptosis.

Although lncRNAs have been shown to have vital biological functions in various malignant tumors, their precise regulatory mechanisms remain largely unknown, although many studies have focused on lncRNA-mediated effects on cell apoptosis. For instance, lncRNA MEG3 inhibits non-small cell lung cancer (NSCLC) cell proliferation and induces apoptosis by affecting p53 expression [31], and lncRNA ANRIL promotes NSCLC cell proliferation and inhibits apoptosis by silencing KLF2 and P21 expression [32]. However, the most important pathway discovered so far is the activation of specific caspase cleavage cascades.

To further confirm the role of caspase activation in Loc554202 induced apoptosis, we found that pretreatment of cells with the pan-caspase inhibitor, Z-VAD-FMK, decreased the Loc554202 induced apoptosis rate, as detected by flow cytometry. Likewise, the outcomes of qRT-PCR and western blot analyses showed that the mRNA and the protein levels of the pro-apoptotic proteins were significantly increased in pCDNA-Loc554202 treated cells, whereas the anti-apoptotic protein was decreased. These data indicate that Loc554202 induces CRC cell apoptosis at least partly through the activation of specific caspase cleavage cascades.

In summary, we have shown that Loc554202 is downregulated in colorectal cancer tissues, and we provided the first evidence that Loc554202 exerts critical effects on CRC cells by affecting both the cell cycle and apoptosis. In addition, CpG island methylation plays an important role in silencing the Loc554202 gene. Finally, we showed that Loc554202 regulated cell apoptosis at least partly through the activation of specific caspase cleavage cascades. Together, our findings suggest that lncRNA Loc554202 acts as a tumor-inhibiting factor in CRC, and could be a candidate prognostic biomarker or a target for new cancer therapies. However, further studies in a larger number of samples and investigations of the other possible mechanisms of action are required.

## Additional files

Additional file 1:
**Sequence of primers. **(XLSX 11 kb) Additional file 2:
**Sequence of si-RNA.** (XLSX 10 kb) Additional file 3: Figure S1. The relative expression levels of miR-31 following the treatment of HCT116 and DLD1 cells with pCDNA-Loc554202 and empty vector. (TIF 3, 271 kb)

## Abbreviations

lncRNAs: Long noncoding RNAs
qRT-PCR: Quantitative reverse transcriptase Polymerase Chain Reaction
HOTAIR: HOX transcript antisense RNA
SPRY4-IT1: SPRY4 intronic transcript 1
PRC2: Polycomb Repressive Complex 2
ZNF703: Zinc finger 703
TNM: Tumor-node-metastasis
DMEM: Dulbecco’sModified Eagle’s Medium
FBS: Fetal bovine serum
siRNA: Small interfering RNA
MTT: 3-(4,5-Dimethylthiazol-2-yl)-2,5-diphenyltetrazolium bromide
PI: Propidium iodide
PBS: Phosphate buffer saline
TUNEL: Terminal deoxynucleotidyl transferase-mediated dUTP nick end labeling
