# Supplementary figures and images for: Long non-coding RNA Loc554202 induces apoptosis in colorectal cancer cells via the caspase cleavage cascades
Source: J Exp Clin Cancer Res. 2015 Sep 11;34(1):100. doi: 10.1186/s13046-015-0217-7 (PMC4567799; doi:10.1186/s13046-015-0217-7)

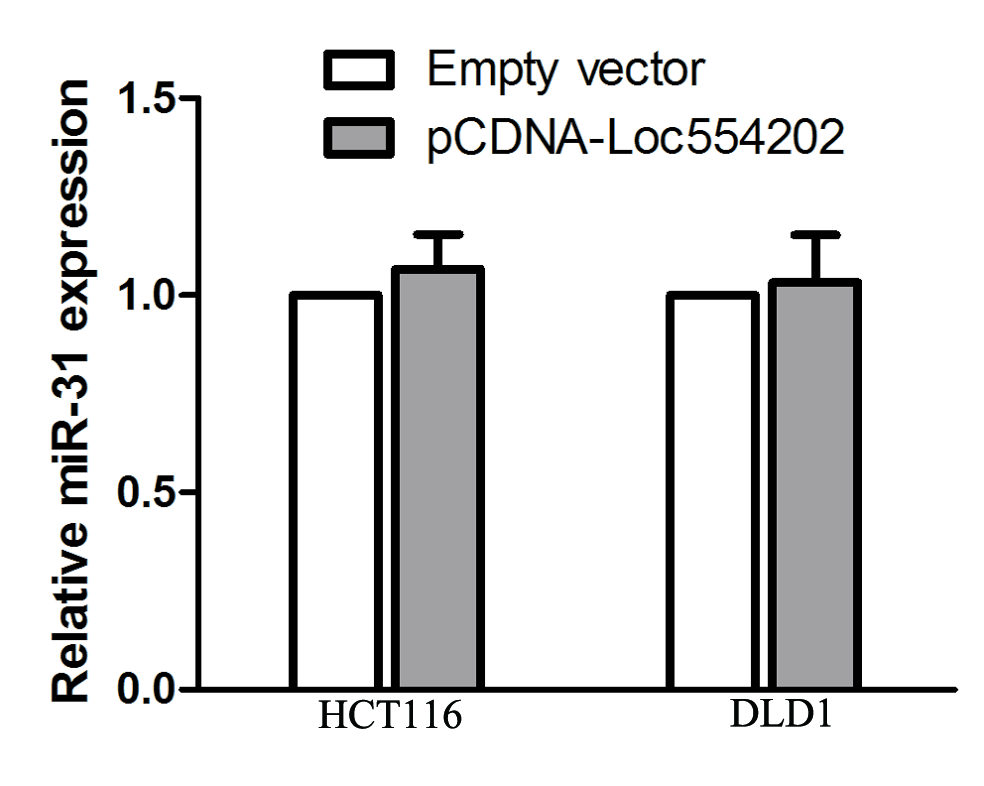

Supplement: Additional file 3: Figure S1. — The relative expression levels of miR-31 following the treatment of HCT116 and DLD1 cells with pCDNA-Loc554202 and empty vector. (TIF 3, 271 kb) [file 13046_2015_217_MOESM3_ESM.tif]
